# Clinical experience with de-escalated chemotherapy for a subset of high-risk gestational trophoblastic neoplasia at a single institution

**DOI:** 10.1007/s10147-026-03105-z

**Published:** 2026-06-23

**Authors:** Kosuke Yoshida, Taito Yoshida, Mari Shirasaki, Yuko Yasui, Eiko Yamamoto, Kaoru Niimi, Hiroaki Kajiyama

**Affiliations:** 1 Department of Obstetrics and Gynecology, Nagoya University Graduate School of Medicine, Tsuruma-cho 65, Showa-ku, Nagoya, 466-8550 Japan; 2 Department of Healthcare Administration, Nagoya University Graduate School of Medicine, Nagoya, Japan

**Keywords:** Gestational trophoblastic neoplasia, Choriocarcinoma, Hydatidiform mole, Invasive, FIGO scoring system, High-risk GTN, Drug therapy

## Abstract

**Background:**

The FIGO scoring system is a well-established method for classifying patients with gestational trophoblastic neoplasia (GTN) worldwide, and the Japanese scoring system is widely employed in Japan. This study aimed to evaluate the clinical outcomes of de-escalated chemotherapy in patients with high-risk GTN, classified as “clinical invasive mole” according to the Japanese scoring system.

**Methods:**

We retrospectively reviewed 268 patients with GTN who received initial treatment at Nagoya University Hospital between 1991 and 2023. All patients were assessed using the FIGO and Japanese scoring systems, and treatment strategies were determined primarily based on histopathological diagnosis and subsequently guided by the Japanese scoring system. Clinical features and treatment outcomes were analyzed.

**Results:**

Among 268 patients, 49 were classified as having high-risk GTN. All patients with FIGO scores of ≥ 9 were classified as “clinical choriocarcinoma” by the Japanese scoring system. In contrast, 17 patients with FIGO scores of 7–8 were divided into the invasive mole group (n = 7) and the choriocarcinoma group (n = 10). All patients in the invasive mole group received de-escalated chemotherapy and achieved complete remission. The proportions of patients with age ≥ 40 years (*p* = 0.046), antecedent hydatidiform mole (*p* = 0.021), shorter interval from index pregnancy (*p* = 0.002), and larger tumor size (*p* = 0.003) were significantly higher in the invasive mole group than in the choriocarcinoma group.

**Conclusion:**

De-escalated chemotherapy may be feasible for select patients with FIGO scores of 7–8, and further refinement of risk stratification is warranted to avoid overtreatment.

**Supplementary Information:**

The online version contains supplementary material available at 10.1007/s10147-026-03105-z.

## Introduction

Gestational trophoblastic disease (GTD) encompasses a heterogeneous group of disorders characterized by the abnormal proliferation of trophoblasts [1–3]. Hydatidiform mole is the most common form of GTD, and gestational trophoblastic neoplasia (GTN) comprises the malignant forms of GTD, including invasive mole (IM) and choriocarcinoma (CC) [1–3]. GTN can develop after any type of pregnancy, including molar pregnancy, abortion, and term delivery [4]. Histological confirmation of CC and IM would require hysterectomy. However, most patients with these diseases are of childbearing age and wish to preserve fertility. Given their high chemosensitivity and frequent metastasis to the lungs, hysterectomy is not the first choice of treatment [3]. Furthermore, reflecting trophoblastic proliferation, serum levels of human chorionic gonadotropin (hCG) are regarded as a highly reliable biomarker for diagnosis and monitoring of GTN [2, 3]. Therefore, the International Federation of Gynecology and Obstetrics (FIGO) scoring system was developed and widely used worldwide for the clinical diagnosis and risk stratification of GTN without the need for histological confirmation [2].

The FIGO scoring system assigns numerical scores based on several prognostic factors, such as patient age, type of antecedent pregnancy, interval from the index pregnancy, pretreatment serum hCG levels, largest tumor size, sites and number of metastases, and history of prior chemotherapy. Based on the total score, patients are classified into low-risk GTN (score 0–6) or high-risk GTN (score ≥ 7) [1, 2]. Meanwhile, the Japanese choriocarcinoma diagnostic scoring system similarly classifies patients into “clinical IM (score 0–4)” and “clinical CC (score ≥ 5)” using clinical parameters such as antecedent pregnancy, interval from the index pregnancy, metastatic status, and serum hCG levels (Supplementary Table 1) [4, 5]. In general, low-risk and high-risk GTN correspond to “clinical IM” and “clinical CC,” respectively. Based on these classifications, single-agent chemotherapy is recommended for low-risk GTN (“clinical IM”), whereas multi-agent chemotherapy is recommended for high-risk GTN (“clinical CC”) [2, 3, 5–7]. However, classification discordance may occasionally occur due to differences in scoring structure between the FIGO and Japanese scoring systems. In such discordant cases, treatment in Japan is generally guided by the Japanese scoring system. Therefore, patients with high-risk GTN but classified as “clinical IM” have been treated with single-agent chemotherapy in Japan.

In this study, we report the treatment outcomes of de-escalated chemotherapy for a subset of patients with high-risk GTN over a 30-year period in a single institution. These findings may contribute to the further optimization of patient stratification and treatment strategies for GTN.

## Patients and methods

### Patients

We retrospectively reviewed patients with GTN who received their initial treatment at Nagoya University Hospital (Nagoya, Japan) between 1991 and 2023. Clinical data collected included age, antecedent pregnancy, interval from index pregnancy, pretreatment serum hCG level, location and number of GTN lesions, and outcomes. The study protocol was approved by the ethics committee of our institute (approval no.2019–0106). Informed consent was waived using an opt-out approach in accordance with institutional guidelines. In addition, written informed consent was obtained from patients whenever feasible. This study was conducted in accordance with the principles of the Declaration of Helsinki.

### Diagnosis and treatment

Patients who underwent uterine curettage or hysterectomy were diagnosed histologically. In addition, all patients were evaluated using the FIGO and Japanese scoring systems. FIGO scores were retrospectively assigned according to the FIGO 2000 scoring system based on available clinical data. The treatment strategy was determined primarily based on the histopathological diagnosis and subsequently guided by the Japanese scoring system. The differences between the FIGO and Japanese scoring systems are summarized in Supplementary Table 1. Serum hCG levels were monitored weekly using a high-sensitivity assay during treatment. At our institution, remission is defined as a serum hCG level of < 0.5 mIU/mL for more sensitive monitoring. In our practice, IM is managed with methotrexate (MTX) or actinomycin D (ACT-D) monotherapy, followed by two additional cycles of chemotherapy after serum hCG levels decrease below 0.5 mIU/mL. CC is treated with the MEA regimen [8], consisting of MTX, etoposide, and ACT-D, followed by four additional cycles after serum hCG levels decrease below 0.5 mIU/mL.

### Statistical analysis

Statistical analyses were performed using R (ver. 4.2.2) and RStudio (ver. 2023.09.1; RStudio, Boston, MA). Comparisons between the two groups were performed using the Mann–Whitney U test. The threshold for statistical significance was set at *p* < 0.05.

## Results

**Fig. 1:**
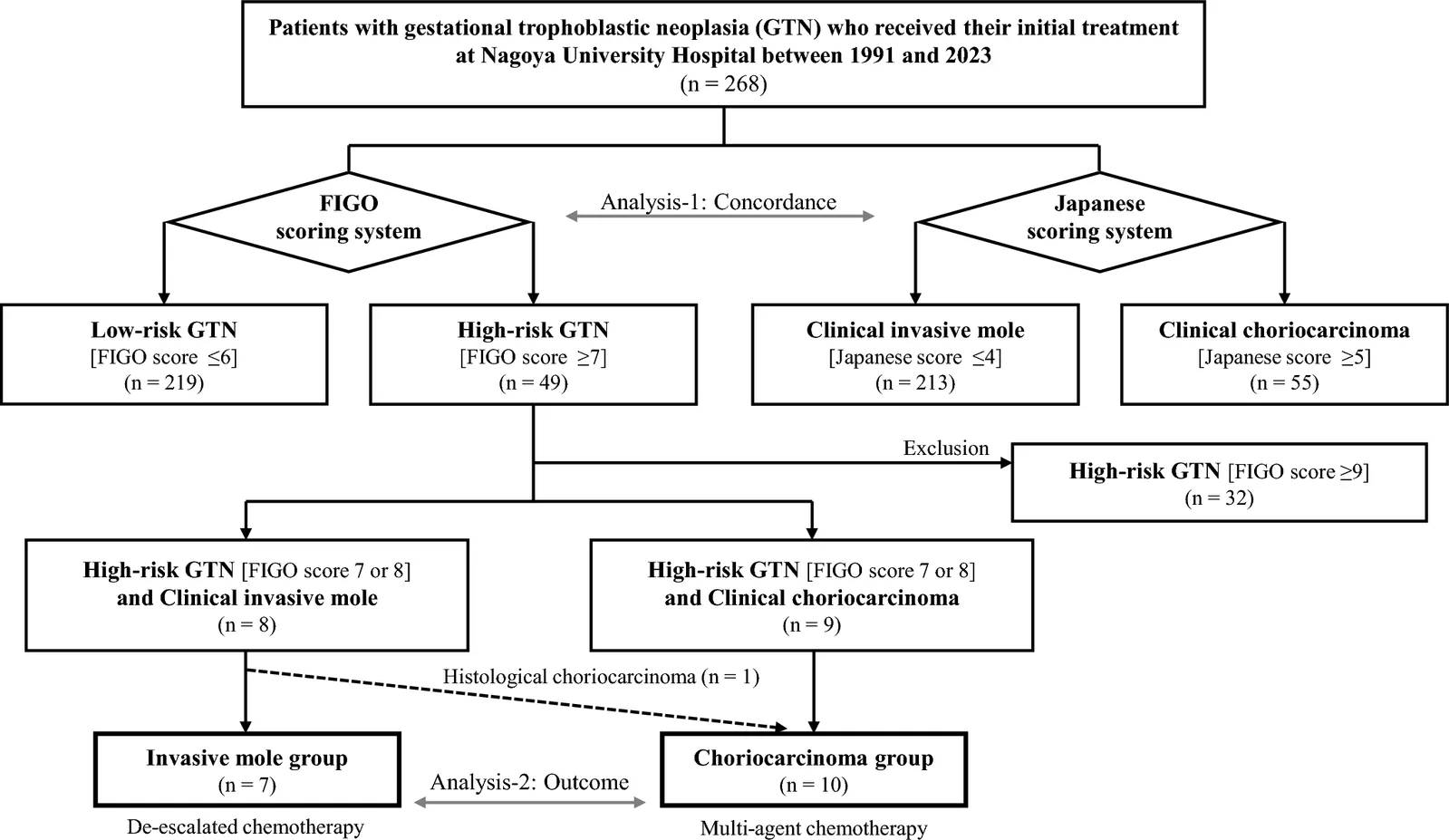
Flowchart of patient selection. Patients with GTN (n = 268) were classified using the FIGO and Japanese scoring systems. Concordance between the two systems was evaluated. Patients with FIGO scores of 7–8 were categorized into clinical IM and CC groups according to the Japanese scoring system, while those with FIGO scores ≥ 9 were excluded from outcome analysis. For outcome analysis, one case initially classified as clinical invasive mole was reclassified as choriocarcinoma based on histological diagnosis. As a result, patients were classified into the invasive mole group (n = 7) and the choriocarcinoma group (n = 10)

In total, 268 patients received their initial treatment for GTN at our institution (Fig. 1). Of these, 219 and 49 patients were diagnosed with low- and high-risk GTN, respectively. According to the Japanese scoring system, 213 and 55 patients were classified as “clinical IM” and “clinical CC”, respectively, resulting in an overall diagnostic concordance rate of 91.8% (246/268 cases; Table 1). In the high-risk GTN group, 24 patients were histologically confirmed as CC and one patient as IM. In the low-risk GTN group, 10 and 21 patients were histologically confirmed as CC and IM, respectively (Table 1).

**Table 1:** Diagnostic concordance between the FIGO and Japanese scoring systems in patients with gestational trophoblastic neoplasia

	Clinical IM* (n = 213)	Clinical CC* (n = 55)
Low-risk GTN (n = 219)	205 [IM (n = 21), CC (n = 4)]†	14 [CC (n = 6)] †
High-risk GTN (n = 49)	8 [IM (n = 1), CC (n = 1)] †	41 [CC (n = 23)] †

^*^ Classified according to the Japanese scoring system for gestational trophoblastic neoplasia

^†^ Cases with histologically confirmed diagnosis

IM, invasive mole; CC, choriocarcinoma; GTN, gestational trophoblastic neoplasia

To investigate the clinical outcome for high-risk GTN patients who received de-escalated chemotherapy, we focused on eight patients with high-risk GTN who were classified as having “clinical IM” according to the Japanese scoring system (Table 1). All eight patients were subsequently found to have FIGO scores of 7 or 8. As a comparison group, nine patients with high-risk GTN (FIGO scores 7–8) who were classified as having “clinical CC” were included (Fig. 1). Patients with FIGO scores ≥ 9 (n = 32) were excluded from this analysis. Detailed patient characteristics are summarized in Supplementary Table S2. Notably, one patient (CC-4) initially classified as “clinical IM” was histologically diagnosed as CC and treated with multiagent chemotherapy. Accordingly, this patient was reclassified into the CC group to reflect the actual treatment strategy. As a result, outcome analyses were performed in the IM group (n = 7) and the CC group (n = 10; Fig. 1).

First, we evaluated the treatment regimens and response to initial therapy. In the IM group, two patients (IM-1 and IM-2) initially received a combination regimen of MTX and ACT-D (MA) at the discretion of the treating physician. After two or three cycles of MA therapy, treatment was switched to ACT-D monotherapy due to elevated levels of liver enzymes. The remaining patients in the IM group received MTX monotherapy. As a result, the response rate to initial treatment was 100% (7 of 7 patients), although all patients were classified as high-risk GTN (Table 2). In contrast, all patients in the CC group received multiagent chemotherapy. Notably, three patients (CC-6, CC-9, and CC-10) had previously received single-agent chemotherapy at referring hospitals because of the diagnostic difficulty of GTN (Supplementary Table S2). Even with multiagent therapy, the response rate to initial treatment in the CC group was 80% (Table 2).

**Table 2:** Clinical outcomes for high-risk GTN cases with FIGO scores of 7 or 8

Group	Patient no	Initial treatment	Secondary treatment	Response rate to initial treatment
IM	IM-1 and -2	MA → ACT-D*	–	100% (7/7)
	IM-3, -4, -5, -6, and -7	MTX	–	
CC	CC-2	MAC → MA*	–	80% (8/10)
	CC-3, -7, -9, and -10	MEA	–	
	CC-4, -6, and -8	MEA → EA*	–	
	CC-1	MAC	EA	
	CC-5	MEA	FA	

^*^Treatment regimen was de-escalated due to adverse events

IM, invasive mole; CC, choriocarcinoma; MTX, methotrexate; ACT-D, actinomycin D; MA, methotrexate plus actinomycin D; EA, etoposide plus actinomycin D; FA, Fluorouracil plus actinomycin D; MEA, methotrexate, etoposide, plus actinomycin D; MAC, methotrexate, actinomycin D, plus cyclophosphamide

Then, we compared clinical information between the IM and CC groups (Table 3). Based on the FIGO scoring system, patients < 40 years old were significantly more common in the IM group than in the CC group (*p* = 0.046). Regarding antecedent pregnancy, complete hydatidiform mole was significantly more common in the IM group (*p* = 0.021). Moreover, the interval from the index pregnancy was significantly shorter in the IM group than in the CC group (*p* = 0.002). Furthermore, tumor size was significantly larger in the IM group than in the CC group (*p* = 0.003). In contrast, there were no significant differences between the two groups in terms of pretreatment serum hCG levels, number of metastases, or previous failed chemotherapy. Based on the Japanese scoring system, six of the seven patients in the IM group had a total risk score of zero (Supplementary Table S2). Additionally, the distributions of antecedent pregnancy type and interval from the index pregnancy differed significantly between the two groups (*p* = 0.021 and 0.008, respectively; Supplementary Table S3).

**Table 3:** Comparison of clinical characteristics between the invasive mole and choriocarcinoma groups according to the FIGO scoring system

	IM group* (n = 7)	CC group* (n = 10)	*p*-value
**Age**			0.0455
0: < 40	2 (28.6%)	8 (80.0%)	
1: ≥ 40	5 (71.4%)	2 (20.0%)	
**Antecedent pregnancy**			0.0208
0: Complete hydatidiform mole	6 (85.7%)	3 (30.0%)	
1: Abortion	1 (14.3%)	2 (20.0%)	
2: Term	0	5 (50.0%)	
**Interval from index pregnancy (months)**			0.0024
0: < 4	6 (85.7%)	1 (10.0%)	
1: 4– < 7	1 (14.3%)	2 (20.0%)	
2: 7– < 13	0	2 (20.0%)	
4: ≥ 13	0	5 (50.0%)	
**Pre-treatment serum hCG**			0.6200
0: < 10^3^	0	2 (20.0%)	
1: 10^3^– < 10^4^	1 (14.3%)	0	
2: 10^4^– < 10^5^	4 (57.1%)	6 (60.0%)	
4: ≥ 10^5^	2 (28.6%)	2 (20.0%)	
**Largest tumor, including uterus (cm)**			0.0030
0: < 3	1 (14.3%)	9 (90.0%)	
1: 3– < 5	6 (85.7%)	1 (10.0%)	
2: ≥ 5	0	0	
**Site of metastases**			–
None	1 (14.3%)	6 (60.0%)	
0: Lung	6 (85.7%)	4 (40.0%)	
**Number of metastases**			0.0767
0: none	1 (14.3%)	6 (60.0%)	
1: 1–4	1 (14.3%)	1 (10.0%)	
2: 5–8	0	0	
4: ≥ 8	5 (71.4%)	3 (30.0%)	
**Previous failed chemotherapy**			–
None	7 (100%)	10 (100%)	
**FIGO score**			0.4237
7	5 (71.4%)	5 (50.0%)	
8	2 (28.6%)	5 (50.0%)	

^*^ Diagnosed either histologically or according to the Japanese scoring system for gestational trophoblastic neoplasia

IM, invasive mole; CC, choriocarcinoma; FIGO, the International Federation of Gynecologic Oncology

## Discussion

In this study, we demonstrated that high-risk GTN cases with FIGO scores of 7 or 8 could be further stratified into two groups based on the Japanese scoring system. Specifically, patients who developed disease shortly after molar pregnancy, in whom higher scores were mainly attributed to tumor size or number of metastases, may have a favorable prognosis. Although the Japanese scoring system is not widely applicable outside Japan, these findings may provide insights for future refinement of risk classification, including potential optimization of the FIGO system.

The FIGO scoring system is a well-established method for patient stratification in GTN. Similarly, the Japanese scoring system, which shares several risk factors with the FIGO system, has also been widely used in Japan since 1967 [5]. Importantly, the Japanese scoring system is fundamentally designed to estimate the underlying histological diagnosis based on clinical parameters. However, the two systems differ in the risk factors considered and their weighting. In the FIGO scoring system, the maximum score for each risk factor is 4 points, and a total score of ≥ 7 is required to define high-risk GTN. Thus, at least two risk factors must be present, and a combination of several moderately weighted factors may be sufficient to classify a patient as high-risk. In contrast, in the Japanese scoring system, the maximum score for each risk factor is 5 points, and “clinical CC” is defined by a total score of ≥ 5 (Supplementary Table S1). This system places greater emphasis on antecedent term pregnancy and an interval of ≥ 36 months from the index pregnancy. These differences in scoring structure may partly explain our finding that patients in the IM group had higher age, shorter interval from the index pregnancy, and larger tumor size (Table 3). In the FIGO system, both age and tumor size contribute to the total score, whereas these factors are not included in the Japanese scoring system. Therefore, additional points from these factors may shift patients into the high-risk GTN category in the FIGO system, while they may still be classified as clinical IM in the Japanese system. In addition, the scoring weight assigned to the interval from the index pregnancy differs between the two systems, with the FIGO system assigning points even for relatively short intervals, whereas the Japanese scoring system emphasizes only prolonged intervals. These differences may contribute to the observed discrepancy between the two classifications. Therefore, refining risk assessment by incorporating these differences may lead to more effective patient stratification.

Over the past several decades, multiagent chemotherapy regimens have evolved considerably for high-risk GTN. Among these, EMA/CO therapy (etoposide, MTX, ACT-D/cyclophosphamide, and vincristine) has achieved high complete remission rates and has become the most widely used first-line regimen worldwide [9, 10]. In addition, MEA therapy (MTX, etoposide, and ACT-D) is also an effective regimen and has been widely used in Japan [8, 11]. In cases of poor response or resistance, the regimen is switched to second-line therapy, and as a result, the overall survival rate of high-risk GTN remains favorable, at approximately 85–94% [9]. Meanwhile, MTX or ACT-D monotherapy is generally selected for patients with low-risk GTN and has been associated with favorable clinical outcomes. However, approximately 30% of low-risk GTN patients are reported to develop resistance after single-agent chemotherapy [3, 6]. A recent study emphasized that patients with FIGO scores of 5–6, particularly those with metastatic disease, CC histology, or elevated pre-treatment hCG levels (≥ 411,000 IU/L without metastases or ≥ 149,000 IU/L with metastases/CC), have an increased risk of single-agent treatment failure [12]. Therefore, further individualization and optimization of GTN treatment strategies are warranted. In this context, our study suggests that even among patients with FIGO scores of 7–8, there may be a subset of those who can be successfully treated with single-agent chemotherapy. Notably, our institution defines remission as a serum hCG level of < 0.5 mIU/mL, which is lower than the commonly used cutoff of 5 mIU/mL. This stringent cutoff, together with a thorough consolidation therapy, may have contributed to the favorable outcomes observed.

Multiagent chemotherapy is highly effective but is also associated with substantial acute and chronic toxicities. Acute adverse events include myelosuppression, neutropenia, anemia, thrombocytopenia, nausea, vomiting, and diarrhea, which can significantly affect patients’ quality of life during treatment [9, 10]. In addition, long-term risks such as premature ovarian insufficiency and infertility are clinically significant concerns [13]. Furthermore, an increased risk of secondary leukemia has been reported with regimens containing etoposide [14]. Therefore, reducing treatment intensity in appropriately selected patients could help minimize unnecessary adverse events.

This study has several limitations. First, the number of patients was small because high-risk GTN with FIGO scores of 7–8 is relatively uncommon, which might limit the statistical power of our analysis. Therefore, the findings should be interpreted with caution. Our results suggest that, based on the well-established Japanese scoring system, further stratification within patients with FIGO scores of 7–8 may be possible. However, this study should be interpreted as a hypothesis-generating analysis rather than a study designed to establish statistically robust differences between groups, and that they do not provide sufficient evidence to support the clinical acceptability of de-escalated chemotherapy in this patient population. Second, the Japanese choriocarcinoma diagnostic scoring system is not widely used outside Japan, which may limit the direct international applicability of our findings. However, the present findings provide preliminary evidence for additional patient stratification, which may contribute to future refinement of risk classification, including potential optimization of the FIGO system. Third, discrepancies between clinical classification and histopathological diagnosis occasionally occur. Therefore, the accurate diagnosis of GTN can be challenging, and our findings should be interpreted and applied cautiously in clinical practice.

In conclusion, de-escalated chemotherapy may be a potential treatment option for select patients with FIGO scores of 7–8. Further accumulation of cases and refinement of patient stratification may facilitate the optimization of treatment strategies for GTN.

## Supplementary Information

Below is the link to the electronic supplementary material.

Supplementary file 1

Supplementary file 2

Supplementary file 3

## Data Availability

The datasets generated and/or analyzed during the current study are not publicly available due to privacy restrictions but are available from the corresponding author on reasonable request.

## References

[1] Joyce CM, Fitzgerald B, McCarthy TV et al (2022) Advances in the diagnosis and early management of gestational trophoblastic disease. BMJ Med 1(1):e000321. 10.1136/bmjmed-2022-000321 36936581 10.1136/bmjmed-2022-000321 PMC9978730

[2] Soper JT (2021) Gestational trophoblastic disease: current evaluation and management. Obstet Gynecol 137(2):355–370. 10.1097/aog.0000000000004240 33416290 10.1097/AOG.0000000000004240 PMC7813445

[3] Ngan HYS, Seckl MJ, Berkowitz RS et al (2025) Diagnosis and management of gestational trophoblastic disease: 2025 update. Int J Gynaecol Obstet 171(Suppl 1):78–86. 10.1002/ijgo.70275 40631439 10.1002/ijgo.70275 PMC12411817

[4] Yamamoto E, Nishino K, Niimi K et al (2022) Epidemiologic study on gestational trophoblastic diseases in Japan. J Gynecol Oncol 33(6):e72. 10.3802/jgo.2022.33.e72 36047375 10.3802/jgo.2022.33.e72 PMC9634103

[5] Sasaki S (2003) Management of gestational trophoblastic diseases in Japan–a review. Placenta 24(Suppl A):S28-32. 10.1053/plac.2002.0933 12842411 10.1053/plac.2002.0933

[6] Lok C, van Trommel N, Braicu EI et al (2025) Practical guidelines for the treatment of gestational trophoblastic disease: collaboration of the European organisation for the treatment of trophoblastic disease (EOTTD)-European society of gynaecologic oncology (ESGO)-gynecologic cancer intergroup (GCIG)-international society for the study of trophoblastic diseases (ISSTD). J Clin Oncol 43(18):2119–2128. 10.1200/jco-24-02326 40359461 10.1200/JCO-24-02326

[7] Seckl MJ, Sebire NJ, Berkowitz RS (2010) Gestational trophoblastic disease. Lancet 376(9742):717–729. 10.1016/s0140-6736(10)60280-2 20673583 10.1016/S0140-6736(10)60280-2

[8] Matsui H, Suzuka K, Iitsuka Y et al (2000) Combination chemotherapy with methotrexate, etoposide, and actinomycin D for high-risk gestational trophoblastic tumors. Gynecol Oncol 78(1):28–31. 10.1006/gyno.2000.5813 10873405 10.1006/gyno.2000.5813

[9] Ji M, Jiang S, Zhao J et al (2022) Efficacies of FAEV and EMA/CO regimens as primary treatment for gestational trophoblastic neoplasia. Br J Cancer 127(3):524–530. 10.1038/s41416-022-01809-3 35459802 10.1038/s41416-022-01809-3 PMC9345879

[10] Singh K, Gillett S, Ireson J et al (2021) M-EA (methotrexate, etoposide, dactinomycin) and EMA-CO (methotrexate, etoposide, dactinomycin/cyclophosphamide, vincristine) regimens as first-line treatment of high-risk gestational trophoblastic neoplasia. Int J Cancer 148(9):2335–2344. 10.1002/ijc.33403 33210289 10.1002/ijc.33403

[11] Sato S, Yamamoto E, Niimi K et al (2020) The efficacy and toxicity of 4-day chemotherapy with methotrexate, etoposide and actinomycin D in patients with choriocarcinoma and high-risk gestational trophoblastic neoplasia. Int J Clin Oncol 25(1):203–209. 10.1007/s10147-019-01540-9 31520175 10.1007/s10147-019-01540-9

[12] Braga A, Paiva G, Ghorani E et al (2021) Predictors for single-agent resistance in FIGO score 5 or 6 gestational trophoblastic neoplasia: a multicentre, retrospective, cohort study. Lancet Oncol 22(8):1188–1198. 10.1016/s1470-2045(21)00262-x 34181884 10.1016/S1470-2045(21)00262-X

[13] Spears N, Lopes F, Stefansdottir A et al (2019) Ovarian damage from chemotherapy and current approaches to its protection. Hum Reprod Update 25(6):673–693. 10.1093/humupd/dmz027 31600388 10.1093/humupd/dmz027 PMC6847836

[14] Bailly C (2023) Etoposide: a rider on the cytokine storm. Cytokine 168:156234. 10.1016/j.cyto.2023.156234 37269699 10.1016/j.cyto.2023.156234

