# Supplementary material for: Clinical experience with de-escalated chemotherapy for a subset of high-risk gestational trophoblastic neoplasia at a single institution
Source: Int J Clin Oncol. 2026 Jun 23;31(9):1960–5. doi: 10.1007/s10147-026-03105-z (PMC13506626; doi:10.1007/s10147-026-03105-z)
Supplement: Supplementary file 1 [file 10147_2026_3105_MOESM1_ESM.docx]

**Supplementary Table S1. Comparison between the FIGO and Japanese scoring systems**

| **Risk factor** | **FIGO scoring system** | **Japanese scoring system** |
| --- | --- | --- |
| **Age** | 0: <40  1: ≥40 | NA |
| **Antecedent pregnancy** | 0: CHM  1: Abortion  2: Term | 0: CHM  3: Abortion  5: Term |
| **Interval from index pregnancy (months)** | 0: <4  1: 4–<7  2: 7–<13  4: ≥13 | 0: <6  4: 6–<36  5: ≥36 |
| **Pre-treatment serum hCG (mIU/mL)** | 0: <10^3^  1: 10^3^–<10^4^  2: 10^4^–<10^5^  4: ≥10^5^ | 0: <10^6^  1: 10^6^–<10^7^  3: ≥10^7^ |
| **Largest tumor, including uterus (cm)** | 0: <3  1: 3–<5  2: ≥5 | NA |
| **Site of metastases** | 0: Lung  1: Spleen, kidney  2: GI tract  4: Brain, liver | 0: None, Lung, pelvis  5: Extrapelvic sites (excluding lung) |
| **Number of metastases** | 1: 1–4  2: 5–8  4: ≥8 | NA |
| **Prior failed chemotherapy** | 2: Single drug  4: ≥2 drugs | NA |
| **Site of the primary tumor** | NA | 0: Uterine corpus, parametrium, vagina  3: Ovary, fallopian tube  4: Uterine cervix  5: Extrapelvic sites |
| **Lung metastasis** | NA | 0: Diameter <2 cm, Number < 20, and  Uniform size  3: Diameter 2–3 cm  4: Variety in size  5: Diameter ≥3 cm or Number ≥21 |
| **Basal body temperature (menstrual cycle)** | NA | 0: Irregular / Monophasic  5: Biphasic |
| **Diagnosis** | <6: Low-risk GTN  ≥7: High-risk GTN | <4: Clinical invasive mole  ≥5: Clinical choriocarcinoma |

FIGO, the International Federation of Gynecologic Oncology; NA, not assessed; CHM, complete hydatidiform mole; GI, gastro-intestinal; GTN, gestational trophoblastic neoplasia.
