# Supplementary material for: Clinical experience with de-escalated chemotherapy for a subset of high-risk gestational trophoblastic neoplasia at a single institution
Source: Int J Clin Oncol. 2026 Jun 23;31(9):1960–5. doi: 10.1007/s10147-026-03105-z (PMC13506626; doi:10.1007/s10147-026-03105-z)
Supplement: Supplementary file 2 [file 10147_2026_3105_MOESM3_ESM.docx]

|  | **IM group***  **(n = 7)** | **CC group***  **(n = 10)** | ***p*-value** |
| --- | --- | --- | --- |
| **Antecedent pregnancy** |  |  | 0.0208 |
| 0: Complete hydatidiform mole | 6 (85.7%) | 3 (30.0%) |  |
| 3: Abortion | 1 (14.3%) | 2 (20.0%) |  |
| 5: Term | 0 | 5 (50.0%) |  |
| **Interval months from index pregnancy** |  |  | 0.0080 |
| 0: <6 | 7 (100%) | 3 (30.0%) |  |
| 4: 6–<36 | 0 | 4 (40.0%) |  |
| 5: ≥36 | 0 | 3 (30.0%) |  |
| **Pre-treatment serum hCG** |  |  | - |
| 0: <10^6^ | 7 (100%) | 10 (100%) |  |
| **Site of the primary tumor** |  |  | - |
| 0: Uterine corpus, parametrium, vagina | 7 (100%) | 10 (100%) |  |
| **Site of metastases** |  |  | - |
| 0: None, Lung, pelvis | 7 (100%) | 10 (100%) |  |
| **Lung metastases details** |  |  | 0.2574 |
| 0: Diameter <2 cm, Number < 20, and  Uniform size | 7 (100%) | 8 (80.0%) |  |
| 4: Variety in size | 0 | 1 (10.0%) |  |
| 5: Diameter ≥3 cm or Number ≥21 | 0 | 1 (10.0%) |  |
| **Basal body temperature (menstrual cycle)** |  |  | 0.1401 |
| 0: Irregular / Monophasic | 7 (100%) | 7 (70.0%) |  |
| 5: Biphasic | 0 | 3 (30.0%) |  |

**Supplementary Table S3. Comparison of clinical characteristics between invasive mole and choriocarcinoma according to the Japanese scoring system**

* Diagnosed either histologically or according to the Japanese scoring system for gestational trophoblastic neoplasia.

IM, invasive mole; CC, choriocarcinoma
